# IbeR Facilitates Stress-Resistance, Invasion and Pathogenicity of Avian Pathogenic *Escherichia coli*

**DOI:** 10.1371/journal.pone.0119698

**Published:** 2015-03-13

**Authors:** Shaohui Wang, Yinli Bao, Qingmei Meng, Yongjie Xia, Yichao Zhao, Yang Wang, Fang Tang, Xiangkai ZhuGe, Shengqing Yu, Xiangan Han, Jianjun Dai, Chengping Lu

**Affiliations:** 1 Shanghai Veterinary Research Institute, Chinese Academy of Agricultural Sciences, Shanghai, 200241, China; 2 Key Lab of Animal Bacteriology, Ministry of Agriculture, Nanjing Agricultural University, Nanjing, 210095, China; 3 School of Public Health, Fudan University, Shanghai, 200032, China

## Abstract

Systemic infections by avian pathogenic *Escherichia coli* (APEC) are economically devastating to poultry industries worldwide. IbeR, located on genomic island GimA, was shown to serve as an RpoS-like regulator in *rpoS* gene mutation neonatal meningitis *E*. *coli *(NMEC) RS218. However, the role of IbeR in pathogenicity of APEC carrying active RpoS has not yet been investigated. We showed that the APEC IbeR could elicit antibodies in infected ducks, suggesting that IbeR might be involved in APEC pathogenicity. To investigate the function of IbeR in APEC pathogenesis, mutant and complementation strains were constructed and characterized. Inactivation of *ibeR* led to attenuated virulence and reduced invasion capacity towards DF-1 cells, brains and cerebrospinal fluid (CSF) *in vitro* and *in vivo*. Bactericidal assays demonstrated that the mutant strain had impaired resistance to environmental stress and specific pathogen-free (SPF) chicken serum. These virulence-related phenotypes were restored by genetic complementation. Quantitative real-time reverse transcription PCR revealed that IbeR controlled expression of stress-resistance genes and virulence genes, which might led to the associated virulence phenotype.

## Introduction

Extraintestinal pathogenic *E*. *coli* (ExPEC) strains have been implicated in a range of infections in humans and animals such as neonatal meningitis, urinary tract infections, pneumonia, and septicemia. ExPEC is currently categorized as newborn meningitis *E*. *coli* (NMEC), uropathogenic *E*. *coli* (UPEC), avian pathogenic *E*. *coli* (APEC), and septicemia-associated *E*. *coli* based on the original host and clinical symptoms [1–4]. ExPEC possess a range of similar virulence factors such as the aerobactin iron transport system, Ibe proteins (IbeA, IbeB, IbeC), the K1 capsule, and types 1 and P fimbriae [3, 5–10]. Mounting evidence shows that poultry can be a vehicle or a reservoir for *E*. *coli* capable of causing urinary tract infections and newborn meningitis [11]. Thus, studying the zoonotic potential of APEC is necessary.

The genetic island of meningitic *E*. *coli* that contains *ibeA* (GimA) has been identified and shown to contribute to NMEC invasion of brain microvascular endothelial cells [12–14]. GimA is present in NMEC and APEC and has 15 genes that form 4 operons. The last operon (*ibeRAT*) of GimA encodes IbeR, IbeA, and IbeT, which contributes to *E*. *coli* K1 invasion of host cells [12–13, 15]. The roles of *ibeA* and *ibeT* in the invasion process of infection were reported [16–19]. Previous studies suggest that IbeR is an RpoS-like regulator of stationary-phase gene expression related to stress-resistance in NMEC strain RS218, which carries a loss-of-function mutation *rpoS* gene [20]. However, the role of IbeR in the virulence of APEC with active RpoS has yet not been investigated.

In this study, IbeR from APEC DE205B was characterized. The *ibeR* and *ibeR-ibeA* mutant and complementary strains were constructed. The effects of IbeR on the virulence, invasion capacity, environment stress-resistance, specific pathogen-free (SPF) chicken serum resistance and gene expressions were evaluated to understand the precise function of IbeR in APEC pathogenicity.

## Materials and Methods

### Bacterial strains, plasmids and growth conditions

Strains and plasmids used in this study are shown in Table 1. The APEC strain DE205B was isolated from the brain of a duck with septicemia and neurological symptoms. DE205B, which was characterized previously [16, 21–23], was used for mutant construction, infection studies and functional assays. *E*. *coli* DH5α was used for cloning and BL21 (DE3) cells were used for protein expression [24–25]. All *E*. *coli* strains were grown in Luria-Bertani (LB) medium at 37°C with aeration. When necessary, medium was supplemented with ampicillin (100 μg mL^-1^) or kanamycin (50 μg mL^-1^).

**Table 1:** Bacterial strains and plasmids used in this study.

Strain or plasmid	Characteristics	Reference
Strain		
DE205B	O2:K1	[16, 22]
ΔibeR	*ibeR* mutant in DE205B	This study
PΔibeR	ΔibeR with plasmid pUC18	This study
CΔibeR	ΔibeR with plasmid pUC18-ibeR	This study
ΔibeA	*ibeA* mutant in DE205B	[16]
ΔibeRibeA	*ibeR*-*ibeA* double mutant in DE205B	This study
DH5α	F-, Δ(lacZYA-argF)U169, recA1, endA1, hsdR17(rk-, mk+), phoA, supE44, λ-	TIANGEN
BL21 (DE3)	F-, *ompT*, *hsdS (r*_*B*_^*-*^ *m*_*B*_^*-*^ *) gal*, *dcm* (DE3)	TIANGEN
Plasmid		
pMD 18-T Vector	Amp, lacZ	Takara
pET28a (+)	Kan, F1 origin, His tag	Novagen
pET28a-ibeR	pET28a (+) carrying *ibeR* gene	This study
pUC18	Amp, lacZ	Takara
pUC18-ibeR	pUC18 carrying *ibeR* ORF and its putative promoter	This study
pKD46	Amp; expresses λ red recombinase	[26]
pKD4	*Kan* gene, template plasmid	[26]
pCP20	Cm, Amp, yeast Flp recombinase gene, FLP	[26]

### Expression of IbeR, antibody production and immunoblotting

The *ibeR* open reading frame (ORF), was amplified with primers WSH166F and WSH167R with added *Nde*I and *Xho*I recognition sites (Table 2) and subcloned into pET28a (+) vector (Novagen, Madison, WI, USA). The resulting plasmid pET28a-ibeR was transformed into competent *E*. *coli* BL21 (DE3) and IbeR protein was expressed by induction with 1 mM isopropyl-beta-D-thiogalactopyranoside induction. Protein purification, quantitation and antibody production were performed as described previously [16, 21, 27].

**Table 2:** Primers used in this study.

Primer	Sequence (5’ to 3’)^a^	Target gene
WSH103F	TGCCAGCATAATGCTGTGAT	*ibeR*
WSH104R	GTACGGGGATCAAACGATGG	*ibeR*
WSH107F	CTGCAGCTTCGATTGCACGC	Upstream region of *ibeR*
WSH33R	TTGATTTTGCCGTTTCTTCT	Downstream region of *ibeR*
WSH109F	ATCAAGCTTACTGGCATAGCATTCT GATACAAGTTCTGAAAATGACTTGA GTGTAGGCTGGAGCTGCTTC	pKD4
WSH110R	GCATCAAAATGAAACACTGCGATAT TAAAATTCTTTCAATTTGAAAAGCA CATATGAATATCCTCCTTAG	pKD4
WSH166F	AGCCATATGGATATTATTATAATGAATA	*ibeR*
WSH167R	GTGCTCGAGATCTGCATGCTCAACATTT	*ibeR*
WSH130F	GACGAATTCACTCGTATGCCTGTGTTGT	*ibeR*
WSH131R	TCAGGATCCCTAATAACCACATTGGCAT	*ibeR*
k1	CAGTCATAGCCGAATAGCCT	pKD4
k2	CGGTGCCCTGAATGAACTGC	pKD4
dnaE RT-F	ATGTCGGAGGCGTAAGGCT	*dnaE*
dnaE RT-R	TCCAGGGCGTCAGTAAACAA	*dnaE*
ibeR RT-F	CAGGTGGTATGAAGCAGGTATT	*ibeR*
ibeR RT-R	CACGTTGCTCGCTCTCATTA	*ibeR*
lpdA RT-F	GTACCAGAACGCCTGCTGGT	*lpdA*
lpdA RT-R	GCTGATACGCTTGGTGAAGAC	*lpdA*
tufB RT-F	TGGTAGTTGCTGCGACTGAC	*tufB*
tufB RT-R	CCAGTTCCAGCAGCTCTTCG	*tufB*
gapA RT-F	CTGGTCTGTTCCTGACTGACG	*gapA*
gapA RT-R	CCTGGCCAGCATATTTGTCG	*gapA*
aphC RT-F	TGACGTTGCTGACCACTACGA	*aphC*
aphC RT-R	TCAGAGCTGCTGTGCCATGC	*aphC*
katE RT-F	AAGCGATTGAAGCAGGCGA	*katE*
katE RT-R	CGGATTACGATTGAGCACCA	*katE*
osmC RT-F	GCGGGAAGGGAACAGTATCTA	*osmC*
osmC RT-R	CATCGGCGGTGGTATCAATC	*osmC*
sodC RT-F	ATCTGAAAGCATTACCTCCCG	*sodC*
sodC RT-R	TCGCCTTGCCGTCATTATTG	*sodC*
yfcG RT-F	GAGGCGAGAACTACAGCATTG	yfcG
yfcG RT-R	CTATCCGAACGCTCATCACC	yfcG
pqiA RT-F	GTGAAACTGATGGCTTACGGC	*pqiA*
pqiA RT-R	TACAACAGGAGCACGAACGC	*pqiA*
ompA RT-F	GCTGAGCCTGGGTGTTTCCT	*ompA*
ompA RT-R	TCCAGAGCAGCCTGACCTTC	*ompA*
aatA RT-F	CCGTACCCGTGTCGCTGTTAC	*aatA*
aatA RT-R	CAGCATTATCAGCATTGCCACT	*aatA*
iucD RT-F	GCTGGGTAGCAGACGGATAT	*iucD*
iucD RT-R	GCATCACTGCCGATTCTTTA	*iucD*
luxS RT-F	ACGCCATTACCGTTAAGATG	*luxS*
luxS RT-R	AGTGATGCCAGAAAGAGGGA	*luxS*
ibeA RT-F	ATGACGGTGGGAACAAGAGAA	*ibeA*
ibeA RT-R	ATACCCCTATTGAATCCGCAT	*ibeA*
ibeB RT-F	GTTAAATTACCGGCGGGCTT	*ibeB*
ibeB RT-R	GGTCAGGCTGATAGACGGGAA	*ibeB*
ibeT RT-F	AGGTACACTGCCGATGCTGGTTTA	*ibeT*
ibeT RT-R	CCGATGCCCATTAATGCAACACCA	*ibeT*
rpoS RT-F	CAGCCGTATGCTTCGTCTTA	*rpoS*
rpoS RT-R	CGTCATCTTGCGTGGTATCT	*rpoS*

^a^ restriction sites are underlined

For immunoblotting, protein samples were subjected to sodium dodecyl sulfate-polyacrylamide gel electrophoresis (SDS-PAGE) and transferred to polyvinylidene fluoride membranes (Amersham Pharmacia Biotech, Piscataway, NJ, USA) as described previously [16, 21–23]. Anti-IbeR or Anti-DE205B serum was the primary antibody, horseradish peroxidase-conjugated anti-rabbit IgG was the secondary antibody and 3,3'-diaminobenzidine was used as the substrate.

### Bacterial resistance to environmental stress and SPF chicken serum

Bacterial resistance to environmental stress was determined as described previously with some modifications [20]. Bacteria were suspended in PBS and diluted to 5 × 10^7^ colony forming units (CFUs)/mL. For alkali resistance, the bacterial suspension was diluted 1:10 in 100 mM Tris, pH 10.0 and incubated at 37°C for 30 min. For acid resistance, one-tenth volume of the bacterial suspension was added to LB (pH 4.0) or LB (pH 5.0) and incubated at 37°C for 20 min. For high osmolarity stress, bacteria were mixed with an equal volume of 4.8 M NaCl and incubated at 37°C for 1 h. For oxidative stress, bacteria were treated with 10 mM H_2_O_2_ at 37°C for 30 min. After stress exposure, bacteria were diluted in PBS and plated on LB agar. Survival was calculated as the ratio of bacterial number under stress to the bacteria number under nonstress. Survival was compared to DE205B.

Bactericidal assays were in a 96-well plates as described previously with some modifications [28–29]. Briefly, SPF chicken serum was diluted to 5%, 12.5%, 25%, 50%, and 100% in PBS. Bacteria were added at different dilutions and incubated at 37°C for 30 min. Bacteria were counted by plating on LB agar. Heat-inactivated SPF serum was used as control.

### Construction of gene mutant and complementation strains

The isogenic mutants ΔibeR and ΔibeRibeA were constructed using the lambda red recombinase method [26]. A kanamycin resistance cassette was PCR amplified with primers WSH109F and WSH110R (Table 2) and transformed into strain DE205B containing plasmid pKD46. Mutants were screened and confirmed by PCR and sequenced using primers k1 and k2 [26] in combination with primers WSH107F and WSH33R. The kanamycin resistance cassette was cured by transforming with plasmid pCP20 and selecting for a kanamycin-sensitive mutant strain, which were designated as ΔibeR or ΔibeRibeA.

For complementation studies, the *ibeR* operon including its putative promoters was amplified using primers WSH130F and WSH131R and the fragment was subcloned into the pUC18 vector. The resulting plasmid pUC18-ibeR and control vector pUC18 were transformed into mutant strain ΔibeR to generate strains CΔibeR and PΔibeR, respectively. To detect the effect of IbeR on growth rate, the growth kinetics of each strain were determined.

### Bacterial invasion assays

Bacterial invasion assays were performed as described previously [16]. Chicken embryo fibroblast DF-1 cell monolayers were washed with Dulbecco's modified Eagle's medium (DMEM) without fetal bovine serum and cells were infected with bacteria at a multiplicity of infection (MOI) of 100 for 2 h, 37°C under 5% CO_2_. Extracellular bacteria were eliminated by adding DMEM containing gentamicin (100 μg/mL). Monolayers were washed and lysed with 0.5% Triton X-100. Released bacteria were counted by plating on LB agar plates. Negative control wells containing DF-1 cells only were used in all experiments. Assays were performed three times.

### Virulence test

To determine the virulence of wild-type, mutant, and complementation strains, 7-day-old ducks were inoculated intratracheally with bacterial suspensions at 10^7^ CFUs. Bacterial CFUs in the injected inoculums were confirmed by plating on LB agar. Negative controls were injected with PBS. Mortality was monitored until 7 days post infection.

The 50% lethal dose (LD_50_) was determined using mouse models as described previously [16, 21–23]. Imprinting control region (ICR) mice, 8 weeks old, were inoculated intraperitoneally with 0.2 mL bacterial suspension at different CFUs. Bacterial CFUs in the injected inoculum were confirmed by plating on LB agar. Negative controls were injected with PBS. Mortality was monitored until 7 days post infection. LD_50_ results were calculated using the method by Reed and Muench [30].

### Counting of bacteria in organs during systemic infection in a rat neonatal meningitis model

Animal systemic infection experiments determined the colonization and invasion capabilities of each strain as described previously [16, 21–23]. Groups of six 8-week-old ICR mice were infected intraperitoneally with 2.0 × 10^6^ CFUs of bacteria. At 24 h post infection, mice were euthanized and dissected. Organs were homogenized and diluted homogenate was plated onto LB agar to determine the number of bacteria colonizing organs.

The capacities to enter the central nervous system was determined for each strain in a neonatal rat model as described previously with some modifications [31–32]. SPF Sprague-Dawley rat pups, 5 days old, were infected intraperitoneally with a bacterial suspension containing 10^7^ CFUs. At 18 h after bacterial inoculation, blood were obtained by tail veins. The rat were then killed, and cerebrospinal fluid (CSF) was immediately obtained by cisternal puncture. Numbers of bacteria in samples were determined by plating 10-fold serial dilutions onto LB agar. Bacterial penetration across the blood-brain barrier was defined as a positive culture.

### Ethics Statement

All animal experimental protocols were carried out in accordance with guidelines of the Association for Assessment and Accreditation of Laboratory Animal Care International. The animal study protocol was approved by the Animal Care and Use Committee of Nanjing Agricultural University (SYXK(SU)2011–0036), China.

### Quantitative real-time reverse transcription PCR

The RNA was isolated from bacteria using E.Z.N.A. Bacterial RNA kits (Omega Bio-Tek, Beijing, China) according to the manufacturer’s instructions. Contaminating DNA was removed using RNase-free DNaseI (TaKaRa), and cDNA synthesis was performed using PrimeScript RT reagent kits (TaKaRa) according to the manufacturer’s protocol. Quantitative real-time reverse transcription PCR (qRT-PCR) was performed to determine transcription of virulence genes using SYBR *Premix Ex* Taq (TaKaRa) and gene-specific primers (Table 2). Relative gene expression was normalized to the expression of the housekeeping gene *dnaE* using the ΔΔ*Ct* method [33]. PCR efficiency (> 90%) for each gene was verified via standard dilution curves.

### Statistical analysis

Statistical analysis for *in vitro* and *in vivo* experiments used GraphPad Software package (GraphPad Software, La Jolla, CA, USA). One-way ANOVA was used for analysis of invasion assay *in vitro* data. Two-way ANOVA was performed on qRT-PCR results. Animal infection study analysis was performed using the nonparametric Mann-Whitney U-Test. Statistical significance was established at *p* < 0.05.

## Results

### Deletion of IbeR does not affect growth kinetics and motility of APEC

The *ibeR* gene from APEC DE205B was first sequenced and submitted to Genbank (Accession No: JQ767181.1). The *ibeR* gene of APEC DE205B was 1950 bp, which was 99% identical to those of APEC O1, NMEC strains RS218 and IHE3034. Based on the sequence, mutant strains ΔibeR, ΔibeRibeA were generated as described previously [26]. For genetic complementation, the recombinant plasmid pUC18-ibeR was transformed into the mutant strain ΔibeR yielding the complementation strain CΔibeR. No significant growth defect was observed among them during growth in LB medium (data not shown). ΔibeR migration on swarming agar plates was similar to the parental strain, indicating that motility was not affected by IbeR (data not shown).

### IbeR is expressed and triggers antibody production in ducks

**Fig 1:**
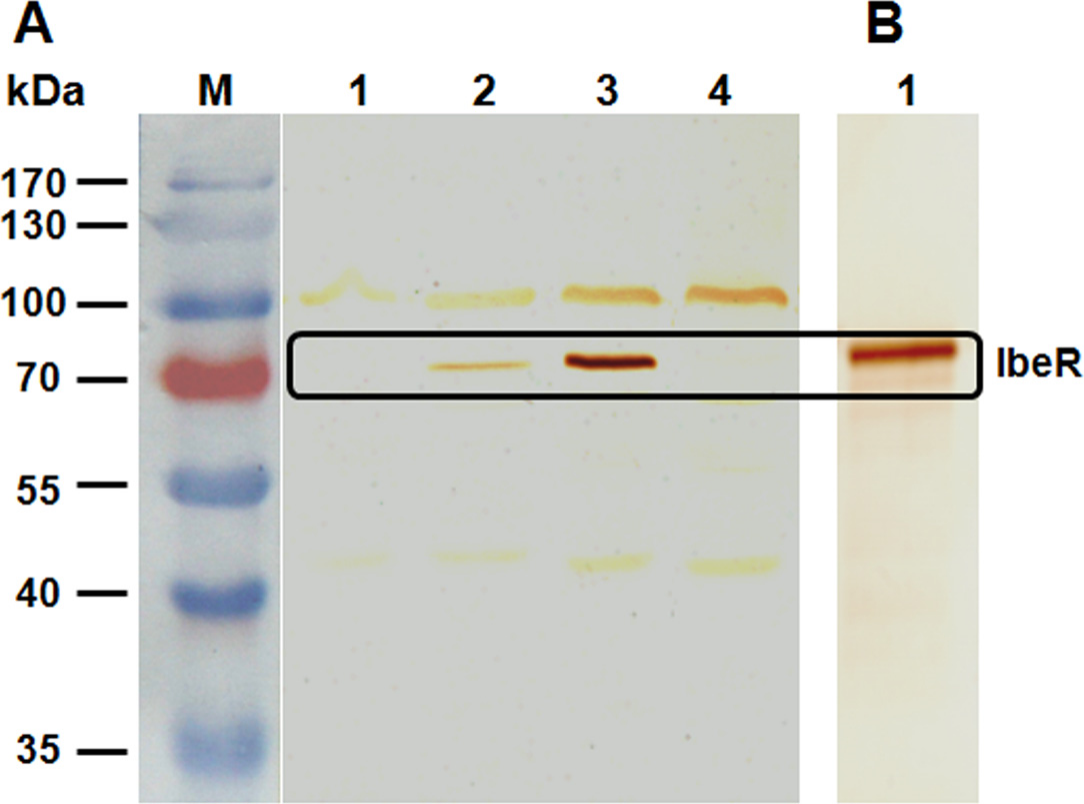
Expression of IbeR by Western blotting. **(A)** Immunoblotting with anti-IbeR of total cell lysates from different APEC strains. Expression of IbeR was detected in wild-type strain DE205B and complementation strain CΔibeR, but not mutant strains ΔibeR or PΔibeR. Lane M, prestained protein marker; Lane 1, ΔibeR (*ibeR* negative); Lane 2, DE205B (*ibeR* positive); Lane 3, CΔibeR (*ibeR* positive); Lane 4, PΔibeR (*ibeR* negative). **(B)** Immunoblotting of purified IbeR protein using anti-DE205B. Incubation with anti-DE205B detected protein bands of the expected size for purified IbeR protein. Lane 1, anti-DE205B.

The expression of IbeR in wild-type, mutant, and complementation strains was compared by SDS-PAGE. No differences in protein patterns between the wild-type and mutant strains were detected (data not shown). Immunoblotting was performed with anti-IbeR serum, showing expected protein bands for IbeR from strains DE205B and CΔibeR. However, no IbeR protein was detected from mutant strain ΔibeR and PΔibeR (Fig. 1). These results indicated that IbeR was expressed under laboratory conditions and verified the construction of the *ibeR* mutant strain.

To determine whether IbeR was expressed and triggered antibody production during infection, immunized anti-DE205B serum was raised. Purified IbeR protein was transferred to membranes and anti-DE205B serum was used as a primary antibody. The results showed that incubation with anti-DE205B led to detected bands of purified IbeR protein, indicating that IbeR elicited an antibody response during infection.

### IbeR is involved in bacterial resistance to environmental stress and serum

**Fig 2:**
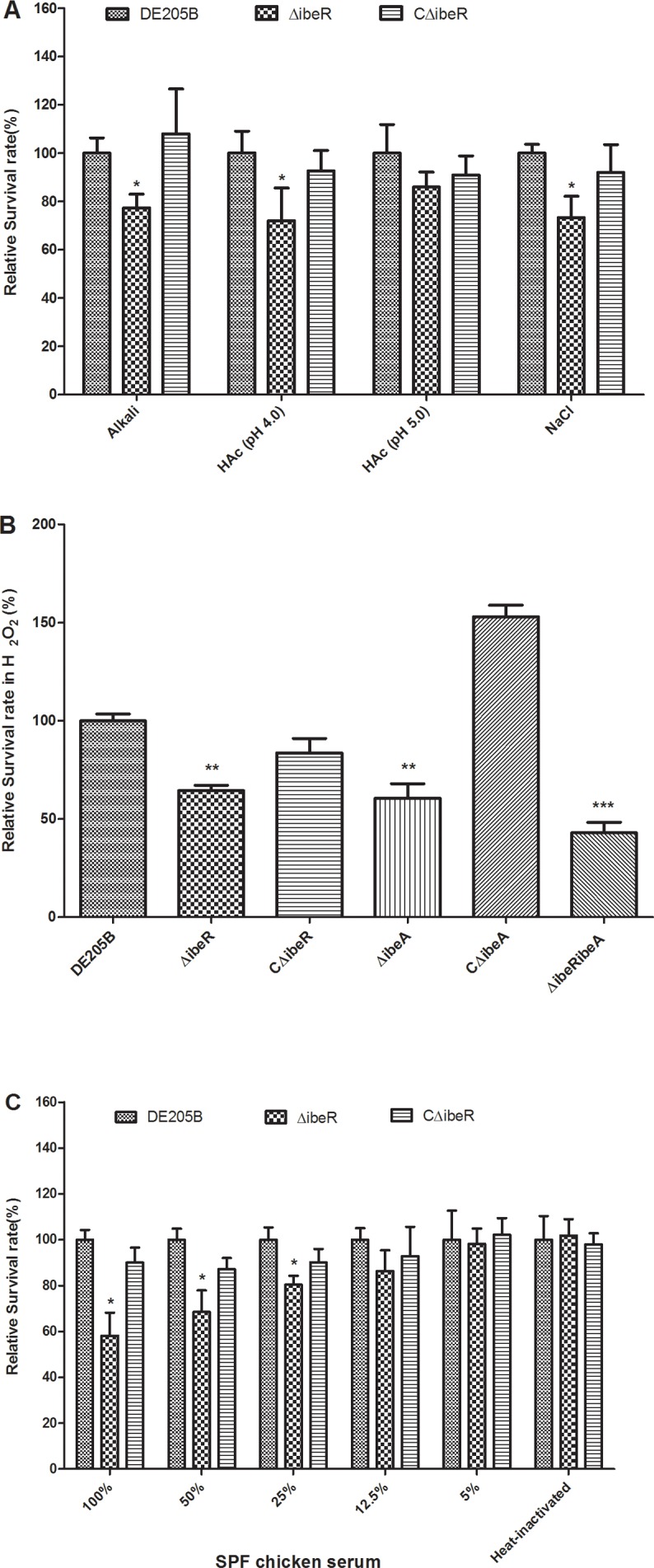
Bacterial resistance to environmental stress and SPF chicken serum. **(A)** Resistance to environmental stress. Each strain was tested for different environmental stress including alkali endurance (pH 10 for 30 min), acid endurance (acetic acid, pH 4.0 and pH 5.0 for 20 min) and high osmolarity challenge (2.4 M NaCl for 1 h). Results were expressed as survival relative to wild-type strain DE205B. Survival of ΔibeR was significantly lower than DE205B (* *p <* 0.05). The complementation strain CΔibeR recovered the most resistance. **(B)** Sensitivity to oxidants of DE205B and its ΔibeR and ΔibeA derivatives. Bacterial suspensions were treated with 10 mM H_2_O_2_ at 37°C for 30 min. After stress exposure, bacteria were diluted in PBS and plated on LB agar. The data were expressed as survival relative to wild-type strain DE205B. Mutant strains ΔibeR, ΔibeA, ΔibeRibeA were more sensitive to H_2_O_2_ killing than the wild type strain DE205B (** *p* < 0.01; *** *p* < 0.001). Moreover, the resistance to H_2_O_2_ was restored for the complementation strains. **(C)** Resistance to SPF chicken serum. Bacteria were incubated at 37°C with SPF chicken serum at different dilutions, and counted at 30 min. Mutant strain ΔibeR showed significantly reduced resistance to SPF chicken serum compared to DE205B (* *p <* 0.05). The error bars indicate standard deviations.

The role of IbeR in bacterial resistance to environmental stresses including alkali endurance (pH 10 for 30 min), acid endurance (acetic acid, pH 4.0 and pH 5.0 for 20 min) and high osmolarity challenge (2.4 M NaCl for 1 h) were determined. In all experiments, survival of wild-type strain DE205B was higher than the mutant strain ΔibeR (Fig. 2A), indicating that IbeR was required for stress-resistance. Previous study showed that GimA and IbeA paly a role in H_2_O_2_ stress-resistance [34]. Thus, the resistance to H_2_O_2_ stress of each strain was determined. The results showed that mutant strains ΔibeR, ΔibeA, ΔibeRibeA were more sensitive to H_2_O_2_ killing than the wild type strain DE205B. Moreover, the resistance to H_2_O_2_ was restored for the complementation strains (Fig. 2B). Thus, our results indicated that the deletion of *ibeR* was responsible for the lower resistance to H_2_O_2_ and other environmental stresses of the mutant strain ΔibeR.

Resistance to serum provides APEC infection and virulence advantages. Bactericidal assays revealed that the mutant strain ΔibeR had lower resistance than the wild-type strain DE205B to SPF chicken serum (*p* < 0.05). Resistance was restored in the complementation strain (Fig. 2C). These results indicated that IbeR was involved in bacterial serum resistance.

### IbeR is necessary for full APEC virulence *in vivo*

**Fig 3:**
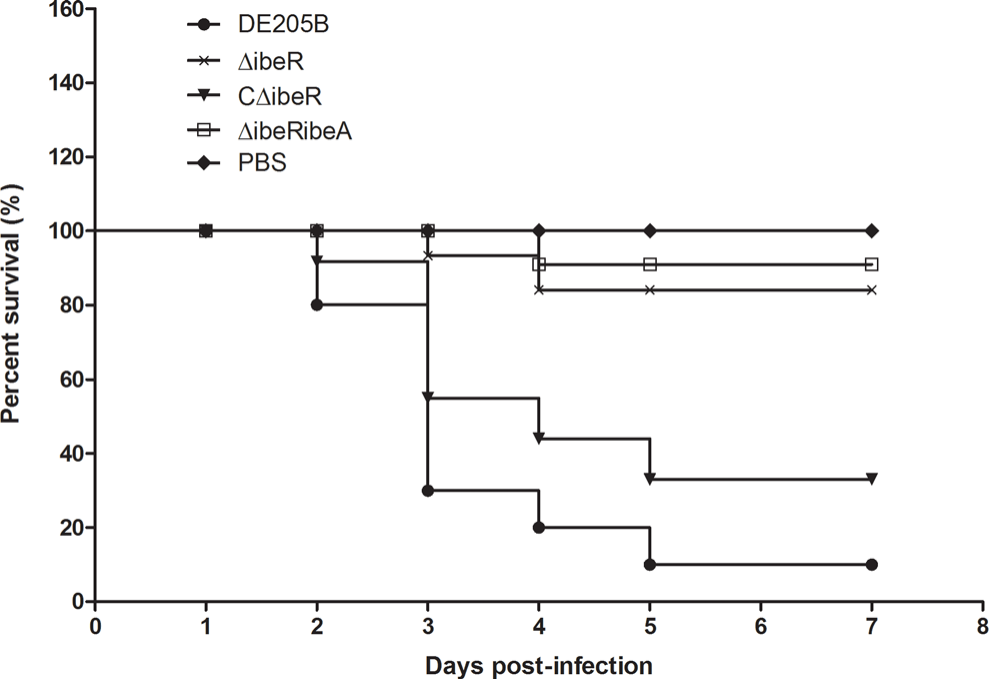
Determination of bacterial virulence. Seven-day-old ducks were inoculated intratracheally with DE205B, ΔibeR, CΔibeR or ΔibeRibeA at 10^7^ colony-forming units (CFUs). Negative controls were injected with PBS. Survival was monitored until 7 days post infection.

To investigate whether IbeR was involved in bacterial virulence, groups of 10 ducks were infected with 1 × 10^7^ CFU of wild-type, mutant, or complementation strains. Mortality was observed for 7 days post challenge. As shown in Fig. 3, the mortality of DE205B, ΔibeR, CΔibeR and ΔibeRibeA were 90%(9/10), 20%(2/10), 70%(7/10) and 10%(1/10), respectively. These results indicated that loss of IbeR or IbeR-IbeA led to attenuation of virulence in birds. Virulence was restored in the complementation strain.

Previous studies indicated that mice and ducks can be used as models to study APEC virulence [16, 21–23]. Thus, the LD_50_ of each strain was evaluated in a mouse model. LD_50_ values were 3.2 × 10^6^ CFU/mouse for ΔibeR and 5.0 × 10^5^ CFU/mouse for DE205B (Table 3). Moreover, the LD_50_ of the complementation strain CΔibeR was partially restored (1.2 × 10^6^ CFU/mouse). These results suggested that IbeR was an important virulence factor in APEC strains.

**Table 3:** Calculations of LD_50_ for different strains.

Dose of challenge (CFU)	No. of dead mice			
	DE205B	ΔibeR	CΔibeR	ΔibeRibeA
2×10^8^	10/10	10/10	10/10	9/10
2×10^7^	10/10	9/10	9/10	8/10
2×10^6^	10/10	3/10	7/10	3/10
2×10^5^	1/10	1/10	1/10	1/10
2×10^4^	0/10	0/10	0/10	0/10
LD_50_ value	5.0×10^5^	3.2×10^6^	1.2×10^6^	5.0×10^6^

### IbeR involvement in APEC invasion of DF-1 cells

**Fig 4:**
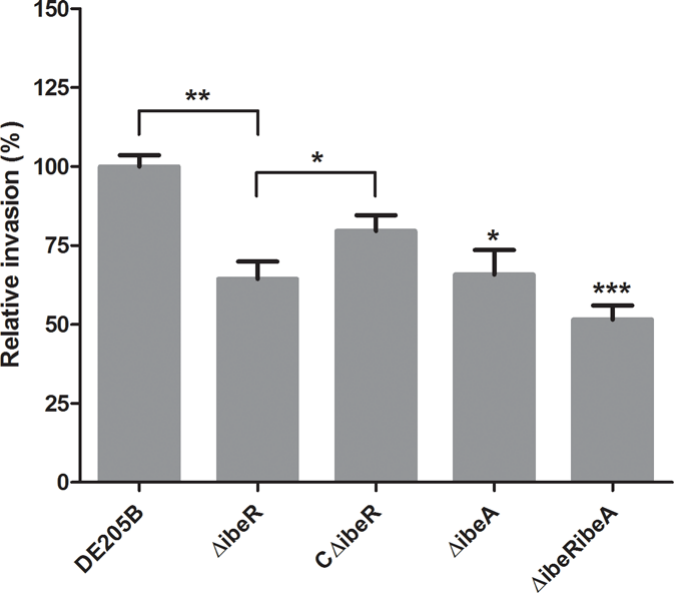
IbeR was involved in invasion DF-1 cells by DE205B. Invasion assays were performed on DF-1 cells. Values are average of three independent experiments. The error bars indicate standard deviations. One-way ANOVA was performed for statistical significance analysis. *** *p* < 0.001; ** *p* < 0.01; * *p* < 0.05.

The role of IbeR in adhesion and invasion of APEC to avian cell lines was determined. The adhesion capacity of mutant strain ΔibeR was similar to the wild-type strain DE205B and the complementation strain CΔibeR, indicating that IbeR did not affect APEC adhesion of DF-1 cells (data not shown). A significant reduction of 35% was detected in invasion of the mutant strain ΔibeR compared with DE205B (*p* < 0.01) (Fig. 4). Invasion capacity was restored in complementation strain CΔibeR, with a significant difference compared to strains ΔibeR (*p* < 0.05). Similar to the results of the virulence test, the double-mutant strain ΔibeRibeA showed decreased ability to invade host cells compared to wild-type and single mutant strains (Fig. 4). Thus, we assumed that IbeR was involved in the invasion of APEC into DF-1 cells.

### IbeR facilitates invasion of APEC during systemic infection and in a rat neonatal meningitis model

**Fig 5:**
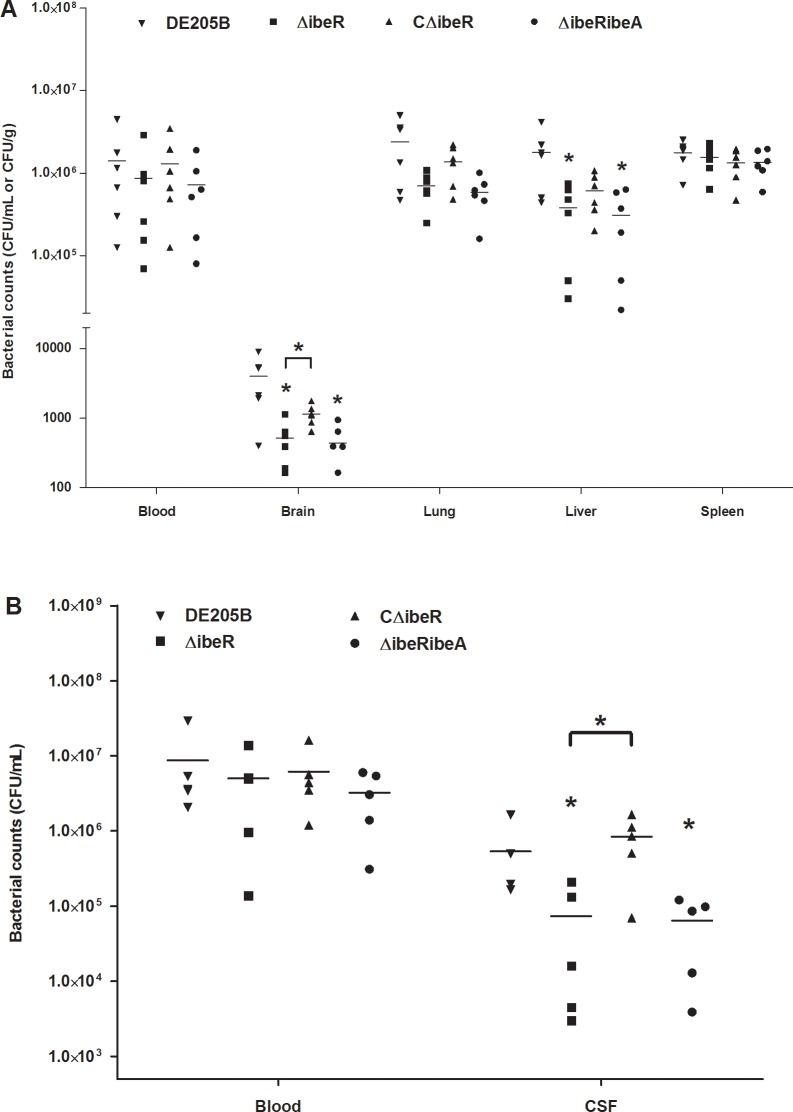
Animal infection experiments. **(A)** Bacterial enumeration during animal systemic infection. Groups of six 8-week-old ICR mice were infected intraperitoneally with a sublethal dose bacterial suspension of 2.0 × 10^6^ CFUs. Bacteria were recovered from blood, brains, lungs, liver and spleen at 24 h post infection. **(B)** Bacterial enumeration in rat neonatal meningitis model. At 5 days of age, groups of five SPF Sprague-Dawley rat pups were inoculated intraperitoneally with a bacterial suspension containing 10^7^ CFUs. At 18 h after bacterial inoculation, blood and cerebrospinal fluid (CSF) specimens were obtained for quantitative cultures. Nonparametric Mann-Whitney U-Test was carried out for statistical significance analysis. * *p* < 0.05.

To determine the effect of IbeR *in vivo*, systemic infection experiments were performed. Bacteria were recovered from blood, brains, lungs, livers and spleens of infected mice at 24 h post inoculation. Recovered ΔibeR compared to wild-type strain DE205B was reduced 1.6-fold in blood, 7.7-fold in brain, 3.4-fold in lung, 4.7-fold in liver, and 1.1-fold in spleen (Fig. 5A). Colonization and invasion capacities in brain and liver were significantly decreased (*p* < 0.05). Recovered complementation strains in organs were restored with differences between strains DE205B and CΔibeR that were not significant (*p* > 0.05). Furthermore, the complementation strain CΔibeR showed significantly increased invasion capacity in brain compared to the mutant strain ΔibeR (*p* < 0.05). These results indicated that IbeR was involved in invasion of the brain of APEC strain DE205B during systemic infection.

IbeR involvement in invasion of host cells was validated in the rat neonatal meningitis model. Using previous methods [31–32], bacteria were recovered from the blood and CSF of infected mice. When mice were infected with ΔibeR, a distinct reduction in numbers of bacterial recovered in CSF was observed compared to DE205B (*p* < 0.05) (Fig. 5B). The invasion capacity of the complementation strain in CSF was restored. Although the recovered bacteria in blood were reduced and restored for strains ΔibeR and CΔibeR, it was not significantly different from the wild-type strain DE205B. These results indicated that IbeR was involved in APEC systemic infection and facilitated invasion into the brain.

### Expression profile of genes involved in resistance and virulence

**Fig 6:**
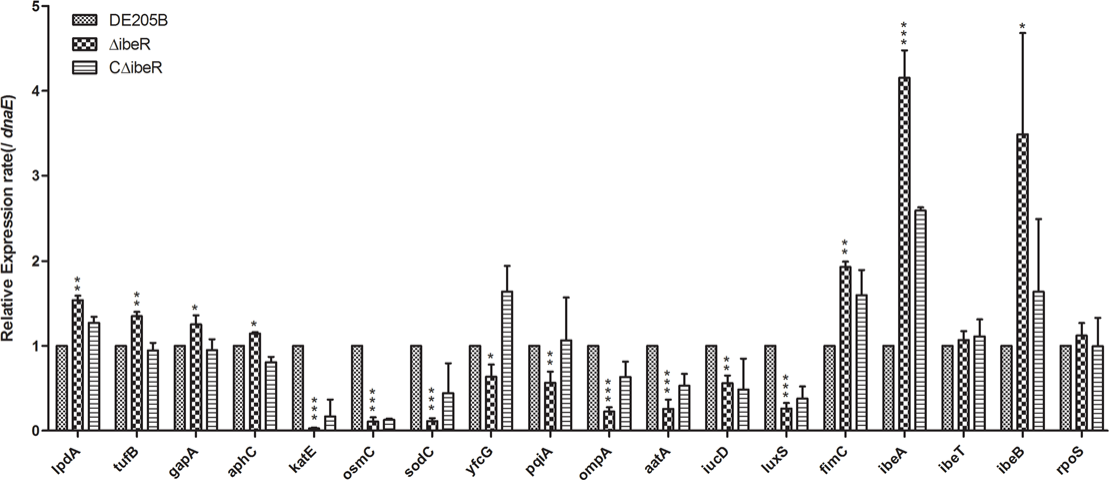
Quantification of gene expression. Expression of genes involved in resistance and virulence were measured by qRT-PCR for each strain. Data were normalized to the housekeeping gene *dnaE*. Results are relative expression ratios compared to wild-type strain DE205B. *** *p* < 0.001; **p < 0.01; *p < 0.05. The error bars indicate standard deviations.

To identify metabolic defaults that was responsible for the decreased resistance and attenuated virulence, the expression levels of range of genes involved in resistance and virulence were analyzed by qRT-PCR for various strains. The results showed that the expression of *lpdA*, *tufB*, *gapA*, *aphC*, *ibeA*, *ibeB* and *fimC* were significantly upregulated in the mutant strain ΔibeR. The mRNA levels were moderately decreased in the mutant strain ΔibeR by 0.23 for *ompA*, 0.26 for *aatA*, 0.56 for *iucD* and 0.26 for *luxS* genes (*p* < 0.01). However, the expression of genes involved in oxidative stress response *katE*, *sodC*, *osmC* were significantly reduced in the mutant strain ΔibeR (Fig. 6).

Previous study showed that IbeR acted as an RpoS-like regulator in NMEC strain RS218, which carries a loss-of-function mutation in *rpoS* gene [20]. Moreover, RpoS is a potential regulator for the expression of some of the genes described above. Therefore, we analyzed whether the deletion of *ibeR* had any influence on the transcript of *rpoS*. Our result indicated that transcription of *rpoS* was not effeced by disruption of IbeR (Fig. 6).

## Discussion

Systemic infections by APEC are economically devastating to the poultry industry worldwide. APEC shares virulence traits with other ExPEC strains (NMEC and UPEC), such as a GimA genomic island. The GimA island consists of 15 genes, some of which are predicted to encode proteins involved in carbon source metabolism and stress-resistance. IbeR, located in GimA, contributes to bacteria stress-resistance in the stationary-phase (SP) in RpoS-negative strain NMEC RS218 [20]. However, the role of IbeR in the strains with active RpoS function is still unknown. Thus, the *ibeR* mutant of APEC DE205B was constructed and characterized. Our results indicated that IbeR acted as a regulator controlling gene expression critical for stress-resistance, and also regulating the virulence genes (*ompA*, *aatA*, *iucD*, *luxS*) for full virulence in the APEC strain with active RpoS.

Expressed APEC IbeR elicited antibodies in infected ducks. Moreover, IbeR controlled gene expression critical for stress-resistance, cell survival and virulence. Thus, we propose that mutation of IbeR results in a decrease in APEC virulence. Animal experiments showed that *ibeR* mutant virulence was decreased compared to the parent strain in duck and mouse models. The complementation strain recovered bacterial virulence. Moreover, the mutant strain did not exhibit a growth defect. Thus, we concluded that IbeR was necessary for full APEC DE205B virulence.

Microbial pathogenicity is a complex phenomenon encompassing diverse mechanisms. However pathogenic organisms use several common strategies such as colonization and invasion to sustain themselves and overcome host barriers. We determined IbeR influence on the virulence and infection of APEC *in vivo* and *in vitro*. Adhesion assays indicated that IbeR was not involved in adhesion of APEC to DF-1 cells. Invasion capacity *in vitro* and *in vivo* of the mutant strain ΔibeR was significantly reduced compared to wild-type strain. Moreover, invasion capacity was restored in complementation strains. These data indicated that IbeR mediated APEC invasion and infection.

APEC infects poultry by initial respiratory tract colonization followed by systemic spread. Resistance to the bactericidal effects and the capacity of APEC strains to cause septicemia and mortality are correlated [35–36]. The capacity to resist serum and environmental stress is an advantage in APEC infection. During infection, the lung environment presents a high oxygen tension, which could lead to a higher rate of production of reactive oxygen species. The bactericidal assays demonstrated that resistance to environmental stress and SPF chicken serum were impaired in the mutant strain ΔibeR (Fig. 3). To determine the metabolic defaults responsible for these phenomenons, we measured the effects of the *ibeR* deletion on the expression of genes involved in the environmental resistance. It has been reported that RpoS regulates *katE*, *sodC*, *osmC*, and *yfcG* gene expression and OxyR regulates *ahpC* genes [34]. Previous study proposed that IbeR acted as a functional equivalent of RpoS in RS218 that presents a loss-of-function mutation in the *rpoS* gene [20]. Our results demonstrated that the *rpoS* was not affected by the *ibeR* mutation. Moreover, the motility, a phenotype linked to RpoS, was not changed in the *ibeR* mutant. Thus, IbeR was resposible for the modification of gene expression and reduced resistance in APEC DE205B carring a active RpoS.

We also measured the effects of the *ibeR* deletion on the expression of virulence genes. The results showed that virulence genes associated with adhesion and invasion (*aatA* and *ompA*) [22, 37], iron acquisition (*iucD*) [11], and quorum sensing (*luxS*) [38], were significantly decreased in the mutant strain ΔibeR compared with DE205B (*p* < 0.05). This expression pattern might be responsible for the reduction of invasion capacities and attenuated virulence of mutant strain ΔibeR. However, the downstream gene of *ibeR*, invasion-associated gene *ibeA*, was significantly upregulated in mutant strain ΔibeR. In this study, lambda red recombinase method was used for the construction of mutant strains, which was used to create nonpolar gene deletion [26]. Moreover, the scar of FRT site did not contain promoter sequence. Then, a *ibeR*-*ibeA* mutant strain was constructed and characterized. Similar to invasion phenotypes of other double (ΔibeA/ΔibeB, ΔompA/ΔibeB) and triple knockouts [39], our experiments revealed that invasion capacity and virulence of the mutant strain ΔibeRibeA were reduced compared to wild-type and single-gene mutant strains. Thus, the reason for increased expression of *ibeA* might be to compensate during invasion for the deletion of IbeR.

In summary, our results demonstrated that IbeR acted as a regulator controlled gene expression critical for stress-resistance genes and virulence genes, which led to impaired resistance to environmental stress and reduced invasion capacity and defective virulence in the active RpoS APEC strain DE205B. The substrate interact with IbeR should to be identified and deserves further study in the future.

## Supporting Information

S1 Table Data for the Figs. 2–5. (XLS)

## References

[1] Johnson JR, Russo TA. Uropathogenic *Escherichia coli* as agents of diverse non-urinary tract extraintestinal infections. J Infect Dis. 2002;186(6):859–864. 12198625 10.1086/342490

[2] Kim KS. *E*. *coli* invasion of brain microvascular endothelial cells as a pathogenetic basis of meningitis. Subcell Biochem. 2000;33:47–59. 10804851 10.1007/978-1-4757-4580-1_3

[3] Dho-Moulin M, Fairbrother JM. Avian pathogenic *Escherichia coli* (APEC). Vet Res. 1999;30(2–3):299–316. 10367360

[4] Ewers C, Janssen T, Wieler LH. Avian pathogenic *Escherichia coli* (APEC). Berl Munch Tierarztl Wochenschr. 2003;116(9–10):381–395. 14526468

[5] Bahrani-Mougeot FK, Buckles EL, Lockatell CV, Hebel JR, Johnson DE, Tang CM, et al Type 1 fimbriae and extracellular polysaccharides are preeminent uropathogenic *Escherichia coli* virulence determinants in the murine urinary tract. Mol Microbiol. 2002;45(4):1079–1093. 12180926 10.1046/j.1365-2958.2002.03078.x

[6] Gunther NWt, Snyder JA, Lockatell V, Blomfield I, Johnson DE, Mobley HL. Assessment of virulence of uropathogenic *Escherichia coli* type 1 fimbrial mutants in which the invertible element is phase-locked on or off. Infect Immun. 2002;70(7):3344–3354. 12065472 10.1128/IAI.70.7.3344-3354.2002 PMC128061

[7] Hoffman JA, Wass C, Stins MF, Kim KS. The capsule supports survival but not traversal of *Escherichia coli* K1 across the blood-brain barrier. Infect Immun. 1999;67(7):3566–3570. 10377140 10.1128/iai.67.7.3566-3570.1999 PMC116545

[8] Pourbakhsh SA, Dho-Moulin M, Bree A, Desautels C, Martineau-Doize B, Fairbrother JM. Localization of the in vivo expression of P and F1 fimbriae in chickens experimentally inoculated with pathogenic *Escherichia coli* . Microb Pathog. 1997;22(6):331–341. 9188088 10.1006/mpat.1996.0116

[9] Torres AG, Redford P, Welch RA, Payne SM. TonB-dependent systems of uropathogenic *Escherichia coli*: aerobactin and heme transport and TonB are required for virulence in the mouse. Infect Immun. 2001;69(10):6179–6185. 11553558 10.1128/IAI.69.10.6179-6185.2001 PMC98749

[10] Mobley HL, Jarvis KG, Elwood JP, Whittle DI, Lockatell CV, Russell RG, et al Isogenic P-fimbrial deletion mutants of pyelonephritogenic *Escherichia coli*: the role of alpha Gal(1–4) beta Gal binding in virulence of a wild-type strain. Mol Microbiol. 1993;10(1):143–155. 7968511 10.1111/j.1365-2958.1993.tb00911.x

[11] Ewers C, Li G, Wilking H, Kiessling S, Alt K, Antao EM, et al Avian pathogenic, uropathogenic, and newborn meningitis-causing *Escherichia coli*: how closely related are they? Int J Med Microbiol. 2007;297(3):163–176. 17374506 10.1016/j.ijmm.2007.01.003

[12] Germon P, Chen YH, He L, Blanco JE, Bree A, Schouler C, et al *ibeA*, a virulence factor of avian pathogenic *Escherichia coli* . Microbiology. 2005;151(Pt 4):1179–1186. 15817785 10.1099/mic.0.27809-0

[13] Huang SH, Wass C, Fu Q, Prasadarao NV, Stins M, Kim KS. *Escherichia coli* invasion of brain microvascular endothelial cells in vitro and in vivo: molecular cloning and characterization of invasion gene *ibe10* . Infect Immun. 1995;63(11):4470–4475. 7591087 10.1128/iai.63.11.4470-4475.1995 PMC173636

[14] Kim KS. *Escherichia coli* translocation at the blood-brain barrier. Infect Immun. 2001;69(9):5217–5222. 11500388 10.1128/IAI.69.9.5217-5222.2001 PMC98628

[15] Prasadarao NV, Wass CA, Huang SH, Kim KS. Identification and characterization of a novel Ibe10 binding protein that contributes to *Escherichia coli* invasion of brain microvascular endothelial cells. Infect Immun. 1999;67(3):1131–1138. 10024553 10.1128/iai.67.3.1131-1138.1999 PMC96439

[16] Wang S, Niu C, Shi Z, Xia Y, Yaqoob M, Dai J, et al Effects of *ibeA* deletion on virulence and biofilm formation of avian pathogenic *Escherichia coli* . Infect Immun. 2011;79(1):279–287. 10.1128/IAI.00821-10 20974831 PMC3019902

[17] Huang SH, Chen YH, Kong G, Chen SH, Besemer J, Borodovsky M, et al A novel genetic island of meningitic *Escherichia coli* K1 containing the *ibeA* invasion gene (GimA): functional annotation and carbon-source-regulated invasion of human brain microvascular endothelial cells. Funct Integr Genomics. 2001;1(5):312–322. 11793250 10.1007/s101420100039

[18] Cortes MA, Gibon J, Chanteloup NK, Moulin-Schouleur M, Gilot P, Germon P. Inactivation of *ibeA* and *ibeT* results in decreased expression of type 1 fimbriae in extraintestinal pathogenic *Escherichia coli* strain BEN2908. Infect Immun. 2008;76(9):4129–4136. 10.1128/IAI.00334-08 18591231 PMC2519445

[19] Huang SH, Wan ZS, Chen YH, Jong AY, Kim KS. Further characterization of *Escherichia coli* brain microvascular endothelial cell invasion gene *ibeA* by deletion, complementation, and protein expression. J Infect Dis. 2001;183(7):1071–1078. 11237832 10.1086/319290

[20] Chi F, Wang Y, Gallaher TK, Wu CH, Jong A, Huang SH. Identification of IbeR as a stationary-phase regulator in meningitic *Escherichia coli* K1 that carries a loss-of-function mutation in *rpoS* . J Biomed Biotechnol. 2009;2009:520283 10.1155/2009/520283 19300523 PMC2655632

[21] Wang S, Shi Z, Xia Y, Li H, Kou Y, Bao Y, et al IbeB is involved in the invasion and pathogenicity of avian pathogenic *Escherichia coli* . Vet Microbiol. 2012;159(3–4):411–419. 10.1016/j.vetmic.2012.04.035 22565007

[22] Wang S, Xia Y, Dai J, Shi Z, Kou Y, Li H, et al Novel roles for autotransporter adhesin AatA of avian pathogenic *Escherichia coli*: colonization during infection and cell aggregation. FEMS Immunol Med Microbiol. 2011;63(3):328–338. 10.1111/j.1574-695X.2011.00862.x 22092559

[23] Zhuge X, Wang S, Fan H, Pan Z, Ren J, Yi L, et al Characterization and Functional Analysis of AatB, a Novel Autotransporter Adhesin and Virulence Factor of Avian Pathogenic *Escherichia coli* . Infect Immun. 2013;81(7):2437–2447. 10.1128/IAI.00102-13 23630958 PMC3697619

[24] Davanloo P, Rosenberg AH, Dunn JJ, Studier FW. Cloning and expression of the gene for bacteriophage T7 RNA polymerase. Proc Natl Acad Sci U S A. 1984;81(7):2035–2039. 6371808 10.1073/pnas.81.7.2035 PMC345431

[25] Studier FW, Moffatt BA. Use of bacteriophage T7 RNA polymerase to direct selective high-level expression of cloned genes. J Mol Biol. 1986;189(1):113–130. 3537305 10.1016/0022-2836(86)90385-2

[26] Datsenko KA, Wanner BL. One-step inactivation of chromosomal genes in *Escherichia coli* K-12 using PCR products. Proc Natl Acad Sci U S A. 2000;97(12):6640–6645. 10829079 10.1073/pnas.120163297 PMC18686

[27] Dai J, Wang S, Guerlebeck D, Laturnus C, Guenther S, Shi Z, et al Suppression subtractive hybridization identifies an autotransporter adhesin gene of *E*. *coli* IMT5155 specifically associated with avian pathogenic *Escherichia coli* (APEC). BMC Microbiol. 2010;10:236 10.1186/1471-2180-10-236 20828376 PMC2944236

[28] Gao Q, Xu H, Wang X, Zhang D, Ye Z, Gao S, et al RfaH promotes the ability of the avian pathogenic *Escherichia coli* O2 strain E058 to cause avian colibacillosis. J Bacteriol. 2013;195(11):2474–2480. 10.1128/JB.02074-12 23504015 PMC3676060

[29] Wang S, Dai J, Meng Q, Han X, Han Y, Zhao Y, et al DotU expression is highly induced during in vivo infection and responsible for virulence and Hcp1 secretion in avian pathogenic *Escherichia coli* . Front Microbiol. 2014;5:588 10.3389/fmicb.2014.00588 25426107 PMC4224132

[30] Reed LJ, Muench H. A simple method of estimating fifty percent endpoints. Am J Hyg. 1938;27:493–497.

[31] Peigne C, Bidet P, Mahjoub-Messai F, Plainvert C, Barbe V, Medigue C, et al The plasmid of *Escherichia coli* strain S88 (O45:K1:H7) that causes neonatal meningitis is closely related to avian pathogenic *E*. *coli* plasmids and is associated with high-level bacteremia in a neonatal rat meningitis model. Infect Immun. 2009;77(6):2272–2284. 10.1128/IAI.01333-08 19307211 PMC2687354

[32] Houdouin V, Bonacorsi S, Brahimi N, Clermont O, Nassif X, Bingen E. A uropathogenicity island contributes to the pathogenicity of *Escherichia coli* strains that cause neonatal meningitis. Infect Immun. 2002;70(10):5865–5869. 12228319 10.1128/IAI.70.10.5865-5869.2002 PMC128312

[33] Livak KJ, Schmittgen TD. Analysis of relative gene expression data using real-time quantitative PCR and the 2(-Delta Delta C(T)) Method. Methods. 2001;25(4):402–408. 11846609 10.1006/meth.2001.1262

[34] Flechard M, Cortes MA, Reperant M, Germon P. New role for the *ibeA* gene in H2O2 stress resistance of *Escherichia coli* . J Bacteriol. 2012;194(17):4550–4560. 10.1128/JB.00089-12 22730120 PMC3415484

[35] Mellata M, Dho-Moulin M, Dozois CM, Curtiss R 3rd, Brown PK, Arne P, et al Role of virulence factors in resistance of avian pathogenic *Escherichia coli* to serum and in pathogenicity. Infect Immun. 2003;71(1):536–540. 12496207 10.1128/IAI.71.1.536-540.2003 PMC143143

[36] La Ragione RM, Woodward MJ. Virulence factors of *Escherichia coli* serotypes associated with avian colisepticaemia. Res Vet Sci. 2002;73(1):27–35. 12208104 10.1016/s0034-5288(02)00075-9

[37] Zhang K, Zhao WD, Li Q, Fang WG, Zhu L, Shang DS, et al Tentative identification of glycerol dehydrogenase as *Escherichia coli* K1 virulence factor *cglD* and its involvement in the pathogenesis of experimental neonatal meningitis. Med Microbiol Immunol. 2009;198(3):195–204. 10.1007/s00430-009-0119-4 19597841

[38] Han X, Bai H, Liu L, Dong H, Liu R, Song J, et al The *luxS* gene functions in the pathogenesis of avian pathogenic *Escherichia coli* . Microb Pathog. 2013;55:21–27. 10.1016/j.micpath.2012.09.008 23046700

[39] Kim KS. Strategy of *Escherichia coli* for crossing the blood-brain barrier. J Infect Dis. 2002;186(Suppl 2):S220–224. 12424701 10.1086/344284

